# Implementation Fidelity of the National Malaria Control Program in Burkina Faso

**DOI:** 10.1371/journal.pone.0069865

**Published:** 2013-07-26

**Authors:** Valéry Ridde, Thomas Druetz, Serge Poppy, Seni Kouanda, Slim Haddad

**Affiliations:** 1 University of Montreal Hospital Research Centre (CRCHUM) and School of Public Health, Montreal, Canada; 2 Institut de Recherche en Sciences de la Santé, Ouagadougou, Burkina Faso; Tulane University School of Public Health and Tropical Medicine, United States of America

## Abstract

**Background:**

Every year 40,000 people die of malaria in Burkina Faso. In 2010, the Burkinabè authorities implemented a national malaria control program that provides for the distribution of mosquito nets and the home-based treatment of children with fever by community health workers. The objective of this study was to measure the implementation fidelity of this program.

**Methods:**

We conducted a case study in two comparable districts (Kaya and Zorgho). Data were collected one year after the program’s implementation through field observations (10 weeks), documentary analysis, and individual interviews with stakeholders (n = 48) working at different levels of the program. The analysis framework looked at the fidelity of (i) the intervention’s content, (ii) its coverage, and (iii) its schedule.

**Results:**

The program’s implementation was relatively faithful to what was originally planned and was comparable in the two districts. It encountered certain obstacles in terms of the provision of supplies. Coverage fidelity was better in Kaya than in Zorgho, where many community health workers (CHW) experienced problems with the restocking of artemisinin-based combination therapy and with remuneration for periods of training. In both districts, the community was rarely involved in the process of selecting CHWs. The components affected by scheduling all experienced successive implementation delays that pushed nets distribution and the initial provision of artemisinin-based combination therapies to the CHWs past the 2010 malaria season.

**Conclusions:**

The activities intended by the program were mostly implemented with good fidelity. However, the implementation was plagued by delays that probably postponed the expected beneficial effects.

## Background

It has been estimated that malaria causes 40,000 deaths every year in Burkina Faso [1]. Yet the most effective strategies to fight this scourge–appropriate prevention and rapid treatment with artemisinin-based combination therapy (ACT) [2]–[5]–are widely known. The available data suggests that in 2010 only 27% of pediatric malaria cases were properly treated within 24 hours [6] and that only 34% of children slept under insecticide-treated nets [7].

This is why, in accordance with international recommendations [8], the national authorities decided in 2010, after running a pilot project in three districts, to implement a national program with two components. The preventive component was aimed at reducing exposure among the entire population, with the goal of raising the rate of utilization of long-lasting insecticidal nets (LLIN) to 80% by 2013. After a population census was conducted, the plan was to distribute one LLIN for every two persons. The curative component of the program involved generalizing the strategy of Home Management of Malaria (HMM) by community health workers to treat episodes of malaria in children. The objective was to treat 80% of simple malaria cases with ACT by 2013. Stocks of drugs were to be supplied to the community health workers (CHWs) in every village. CHWs were to be paid a monthly stipend (5,000 F CFA) and were supposed to sell the ACT at subsidized prices (100 F CFA for treatment of a child, 200 F CFA for adolescents, and 300 F CFA for adults), include a 25% profit margin. The plan called for them to be able to restock their supplies of ACT at the nearest community primary care centre (CSPS).

The program received funding of 63 million Euros for five years from the Global Fund to Fight AIDS, Tuberculosis and Malaria. There are two main recipients of this program funding. The first is the State, for the program to support health districts in carrying out the LLIN component, and the second is the non-governmental organization (NGO) PLAN-International, for the community-based home treatment component. The NGO coordinates this component in collaboration with the National Malaria Control Program (NMCP). PLAN has delegated field operations to four national NGOs (considered secondary recipients), as the country’s territory is subdivided into four zones. In the two districts of the present study, the NGOs are the *Union des Religions et des Coutumiers du Burkina* [Union of Burkina religions and tribal chiefs] in Kaya, and ‘*Songtaaba*’ in Zorgho.

The ultimate aim of these two components is to reduce the incidence of malaria and save lives. The effectiveness of this approach has been widely proven in pilot settings or in experimental clinical trials in which implementation conditions were closely controlled and their efficacy could be monitored [9]. On the other hand, it is well known that practical effectiveness can be seriously constrained by an implementation that does not respect what was planned, especially when a program is scaled up nationally. For instance, a systematic review showed the current practical effectiveness of programs to be only about half of the level of effectiveness observed in optimal conditions (efficacy) [10].

Given Burkina Faso’s context, with its public policies and the fact that part of the program was confided to an NGO, there has been some concern that the program’s implementation could deviate quite far from the original plans and that this could generate a so-called implementation gap. The corollary would be the appearance of a sizeable gap between the effectiveness expected of the program based on its efficacy and its practical effectiveness in real-life conditions [11]. Implementation gaps [10], [12] have already been uncovered in Burkina Faso, particularly in malaria control programs [13], [14]. Indeed, we know that the health system is fragile, that the funding and realization of national initiatives depends very much on the commitment of external partners, that district-level leadership is a variable factor in program effectiveness, that the monitoring system is not always reliable, and that health policies are not always implemented as intended [15]–[20].

Even though implementation analysis can, to some extent, explain the success or lack of success of programs that are carried out, it is still rarely done [21], [22], particularly when it comes to the scaling-up of public health programs [23]. Thus, this article reports the results of an implementation evaluation of the program scaled up nationally in Burkina Faso in 2010. The objective was to assess the extent to which the activities were carried out as originally planned.

We were interested in examining the fidelity of the implementation, with the assumption that practical effectiveness would more closely approach theoretical effectiveness to the extent that the activities respected the original program plan in terms of content, coverage, and schedule, which are the factors most frequently cited in fidelity studies [24]–[26]. More specifically, our aim was to verify whether (i) the planned activities were implemented (content), (ii) the number of planned activities and the territory involved were respected (coverage) and, finally, (iii) the planned timetables and sequences of activities were respected (schedule). In the schedule dimension, we combined frequency and duration, from the analysis framework of Carroll et al. and that proposed by Hasson [24], [26].

## Methods

### Methodological Approach

The evaluation was carried out using a multiple case study with several embedded levels of analysis [27]. The case was the program. It was a multiple case, in that we compared its implementation in two districts. The levels of analysis were the three dimensions of implementation fidelity as well as four functions that covered the activities described below. We selected the first district (Kaya) because we have been carrying out an impact analysis of the program there using a demographic surveillance system that provides data on children’s health. The survey site includes 18 villages, the city of Kaya and seven community primary care centres (CSPSs). The second district (Zorgho) was selected because it is the most comparable district in that region, from both the ecosystemic and socioeconomic standpoints (Table 1). It also serves as a comparator district for the impact study we are conducting. The survey site includes 18 villages, the city of Zorgho and five CSPSs.

**Table 1:** Comparison of the two districts studied.

	Kaya District	Zorgho District
Total population	500,008	352,003
Number of cases of malaria per inhabitant per year	0.25	0.38
Annual rainfall	506 mm	661 mm
Percentage of households under the poverty line	44%	41%
Language spoken	90% Moore	89% Moore
Distance from the capital	98 km	103 km

Source: Ministry of Health (2011); Ministry of the Economy and Finances (2009); RGPH (2006).

**Figure 1:**
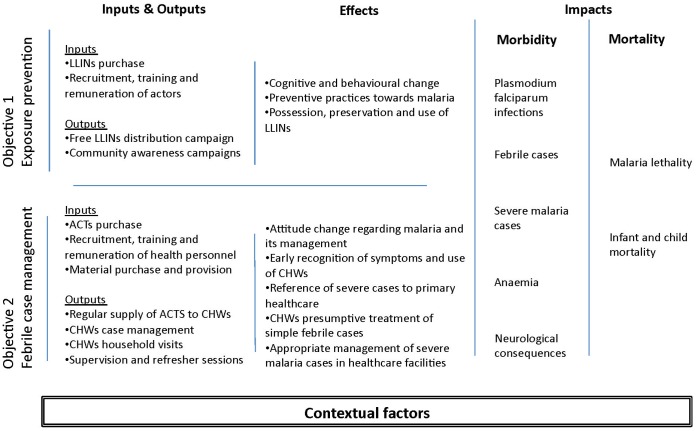
The MEILUP’s intervention theory and causal chain of events.

In the evaluability assessment [28], we described the program’s intervention theory and compiled the list of activities envisioned in the original plan (Figure 1). This list was reconstituted by our team from program planning documents and eight interviews with key stakeholders. They validated this list, which comprised 12 activities for the preventive component (LLIN) and 35 activities for the curative component (HMM). To facilitate the analysis, these 47 activities were grouped into four functions: (i) recruitment and training; (ii) provision of supplies (only for HMM); (iii) remuneration; and (iv) conduct of activities. These functions were derived from the program’s intervention theory – the first three concern its inputs while the fourth correspond to its output. The list of activities is presented in Table S1 and S2.

### Data Collection

The empirical data were drawn from field observations (10 weeks), documentary analysis (five project documents and numerous forms from CHWs and CSPSs), and 48 individual interviews with key stakeholders involved in the program’s implementation (Table 2). The interviews were conducted in French or in Moore, depending on the respondent, and were based on an interview guide that was tested beforehand. The data were collected between June and August 2011, that is, seven (Zorgho) and 14 (Kaya) months after the program’s launch. Of the CHWs encountered, 86% were women (25/ 29), 55% were literate (16/ 29), 44% had become CHWs in the 1980 s (13/29), 10% in the 1990 s (3/29), and 44% since 2000 (13/29). Of the NGO organizers interviewed, 43% were women (3/7). Two of the 11 nurse health-post managers (NHMs) were women.

**Table 2:** Number of interviews carried out in the two districts.

	KAYA	ZORGHO
Community health workers	15	14
Nurse health-post managers	6	5
NGO organizers	3	4
TOTAL	25	23

### Data Analysis

The data were analyzed using a framework analysis process [29], that is, the empirical data were studied in terms of the three dimensions of fidelity.

In analyzing the content of the activities carried out, the data were first used to quantify the proportion of activities that fit within each of five possible modalities that could be used to consider how the actors had reinvented the activities [25]: implemented as intended (I), modified (M), implemented or modified (I or M), added (A), not implemented (NI). Some activities could not be coded in certain CSPSs because there was no one to carry them out (which was mainly the case of one CSPS in Kaya). In contrast to Perez et al. [25], we added the third modality because for some activities our respondents remembered implementing the activities but were not sure they had not been modified (for example, someone would remember being trained or paid, but no longer remembered how many days the training lasted, or what amount was received). The qualitative interviews were then transcribed, coded and organized according to the analysis framework using NVivo**®** software. Syntheses by district and by stakeholder groups provided qualitative data to illustrate the proportions quantifying content fidelity.

To analyze the coverage of activities, we looked at whether the implementation had respected the number of activities planned and the territory involved.

To analyze the schedule, we focused on activities for which dates and time periods had been specified in the planning documents, of which there were ultimately very few.

Drawing upon the work of Perez et al. [25], graphical figures produced with Excel**®** were used to visually present the results related to content fidelity, the details of which are provided in Table S1, S2, S3 and S4. Two of the authors independently and systematically analyzed these proportions using a coding guide. In cases of disagreement, they discussed the analyses until agreement was reached.

The study was authorized by the health research ethics committees of Burkina Faso and of the CRCHUM in Canada. Written consent was obtained for every participant.

## Results

### Content Fidelity

On the whole, the results showed good content fidelity of the activities, although better for the LLIN component than for the HMM component (Table 3). As well, there was no striking difference between the two districts. In Kaya, the HMM component seemed to have experienced some implementation difficulties, while in Zorgho, both components encountered the same problems. Implementation of the LLIN component in Zorgho presented certain specific difficulties compared with Kaya. In both districts, it was only in the HMM component that some activities were added, over and above what had been planned, such as retraining of CHWs.

**Table 3:** Content implementation fidelity.

	KAYA		ZORGHO	
	LLIN	HMM	LLIN	HMM
Implemented as intended	64.0%	53.0%	56.0%	62.5%
Implemented or modified	20.0%	7.5%	13.0%	3.0%
Modified	16.0%	17.0%	15.0%	12.0%
Added	0.0%	7.5%	0.0%	5.0%
Not implemented	0.0%	15.0%	16.0%	17.5%
	**100.0%**	**100.0%**	**100.0%**	**100.0%**

Source: survey data.

**Figure 2:**
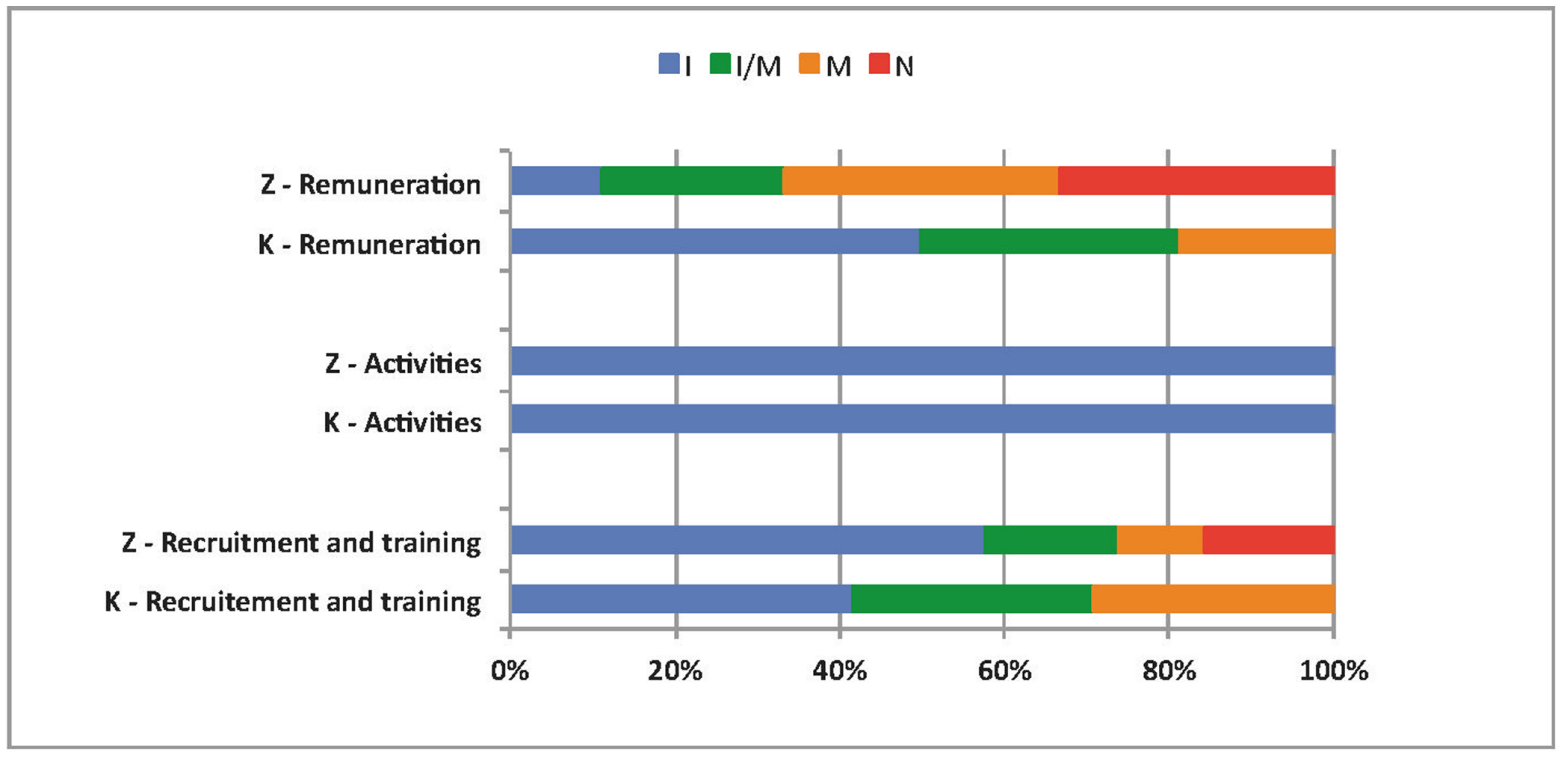
Comparison of the content fidelity of the LLIN component in the two districts. Notes: I = implemented as intended; I/M = implemented or modified; M = modified; N = not implemented; Z = Zorgho; K = Kaya**.** Source: survey data.

**Figure 3:**
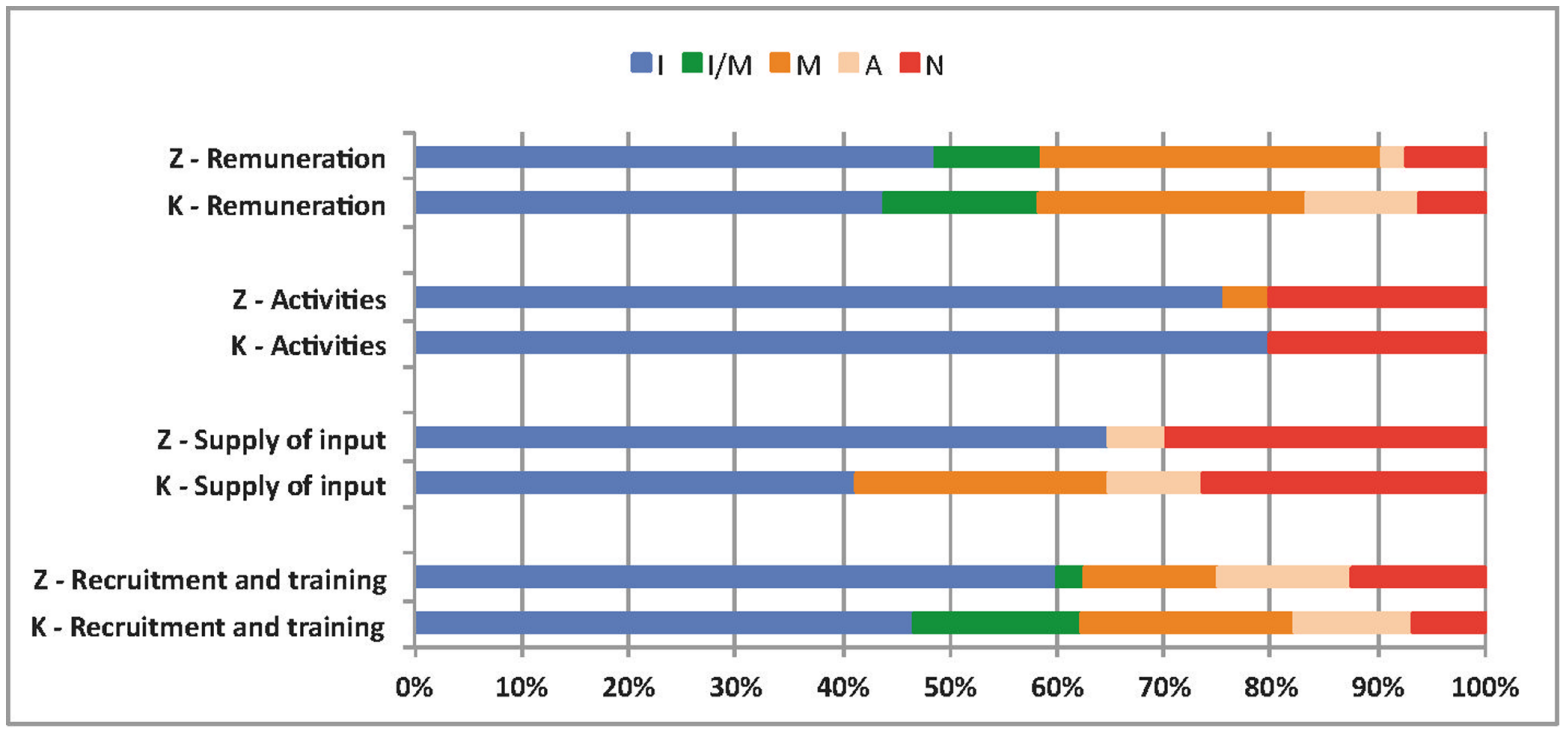
Comparison of the content fidelity of the HMM component in the two districts. Notes: I = implemented as intended; I/M = implemented or modified; M = modified; A = added; N = not implemented; Z = Zorgho; K = Kaya. Source: survey data.

The detailed results for the 47 activities in both districts are provided in Table S1 (Kaya) and S2 (Zorgho). They are summarized in Figure 2 for the LLIN component and Figure 3 for the HMM component.

In general, the 47 activities were not implemented differently in the two districts, except with regard to remuneration. In Zorgho, the major difficulty had to do with remuneration of CHWs and NHMs in four of the five CSPSs. One CHW told us, “*I was trained in one day, and was not remunerated*”; this did not happen in Kaya. In addition, some of the census-taking training activities for the NGO organizers were not implemented in three CSPSs because they were absent when the training occurred. In both districts, all the LLIN distribution activities were conducted as planned. Even so, they were not without incident.

First, taking the population census was difficult because it was done in a period of intense agricultural activity, when people are not very available and not generally at home, and the census was sometimes perceived with suspicion. According to one CHW in Kaya, people feared “*that the census was being done for taxation purposes.*” Then, even though the plan called for distributing one LLIN to every two persons, it was ultimately decided to provide fewer. “*The one thing they didn’t understand was the reduction in the number of LLINs […] people were unhappy about that,*” said a CHW in Zorgho. On top of that, only people who were given an LLIN voucher at the time of the census were supposed to receive nets, but under pressure from those who either had not received a voucher or had lost theirs, the decision was taken to give LLINs even to households with no voucher.

The area in which the original plan was least respected was the provision of supplies. In Zorgho, no CHW had received a consultation register. While they had all received the initial stocks of ACT, in three out of four CSPSs the CHWs were unable subsequently to replenish their stocks. “*We ran out of drugs […] we went to the CSPS and they didn’t have any drugs*,” said a CHW in Zorgho. This problem did not occur in Kaya, because of the multiple interventions going on specifically in that district.

In fact, in Kaya the situation regarding ACT inputs was more complicated because two other interventions had rendered the implementation more complex, such that even the CHWs sometimes became confused when trying to explain their activities to us. First, Kaya was a Round 7 pilot site, so they had already received ACTs and were therefore supposed to replenish their stocks through the sales of those drugs, without any new funding from the program. However, they did not understand this cost-recovery system, and they waited for a project from the Ministry of Health funded by the Bill & Melinda Gates Foundation (BMG) for children under age five that supplied them with ACTs; “*we’re working with BMG ACTs*,” said one CHW. This supply of drugs from BMG was considered a stock replenishment, coming not from the NMCP but from another intervention, which explains why the activity was considered to have been modified from the original plan. The CHWs also did not receive any new consultation registers for children, keeping only the one received by BMG. However, these ACTs were limited to this target clientele of children under five years, whereas in 2010, the HMM program was intended for everyone. Then, these BMG stocks either ran out, as had the HMM stock (“*I put in the order, but the depot told me there wasn’t any*,” said one CHW) or expired (“*what we have here is for children 2 to 11 months, but it’s expired*,” another CHW told us). Indeed, in the case of one CHW working in a rural setting, for example, 197 of 200 packages of ACT for 2 to 11 month-olds received November 1, 2010, were expired at the time of our observation on August 28, 2011. Finally, in July 2011 a new intervention took everyone by surprise. An NGO decided to make all healthcare free for children under five years in the district, such that “*we didn’t put in an order*,” said one CHW, since mothers preferred to go to the CSPS, where ACTs were free, even if it required travel, rather than pay to obtain them from the CHW in their village.

On the other hand, in four out of six CSPSs in Kaya, the CHWs did not receive any carrying cases for the ACTs; thus, one CHW told us, “*I transport my drugs in a cardboard box, since I didn’t get a bag*.” In Zorgho, all the CHWs received from the NGO, not a carrying case as such, but a cloth bag to be used as a carrying case that was stamped with the logos of the program and the Global Fund.

In Zorgho, in only three of the five CSPSs, the CHWs received boxes of images (an added activity) for health education. In Kaya, on the other hand, this component was added without any problem. No NGO organizer in Kaya or Zorgho received the audiovisual material that was in the plan, but they all received motorbikes for their supervision activities.

In both districts, the CHWs reported that most of the activities with families were carried out. The same was true of the follow-up done by the organizers from the two NGOs. On the other hand, the health education skits and film projections planned by the organizers were not carried out.

Likewise, in two of Kaya’s six CSPSs and four out of five of Zorgho’s, the NHMs did not carry out the activity of approving the CHWs’ monthly activity reports. One NHM confirmed that, “*No, I never received any. Maybe the NGO organizer did. I can tell you that the organizer sends us reports, maybe it’s incorporated into those, but I don’t know*.” We also observed on several occasions that the NHMs did not know about activities carried out with and for the CHWs. For example, in Zorgho the NHMs told us the CHWs had not received any case to carry the ACTs, when this was not so. The reason for this was that the cases were distributed directly to the CHWs by the NGO organizers without going through the NHMs. In general, it should be noted that the NHMs had almost no involvement in the program, and no resources appeared to have been planned for them. “*You know, often it’s difficult, because we could go out to do supervision, but nothing is planned. Nothing. We asked for fuel, but they said it wasn’t possible. So, it’s hard, even if you wanted to…,*” said one NHM in Zorgho. However, particularly in Kaya, the NHMs considered that the NGO organizers’ role was useful and helped fight malaria.

### Coverage Fidelity

Table 4 shows that the coverage of activities was more complete in Kaya than in Zorgho. Further details are provided in Table S3 and S4. The LLIN distribution was modified at the national level, since it was the Ministry authorities who issued the directive to reduce the number of LLINs distributed to households. Thus, every individual who arrived with more than two vouchers was given one LLIN less than the number he was supposed to receive. As such, the planned ratio of two persons per LLIN was not respected for households of more than four persons.

**Table 4:** Coverage implementation fidelity.

	KAYA		ZORGHO	
	LLIN	HMM	LLIN	HMM
Recruitment and training	96.0%	76.0%	71.0%	77.0%
Provision of stock	-	70.0%	-	69.0%
Activities	84.0%	83.0%	87.0%	76.0%
Remuneration	97.0%	95.0%	63.0%	76.0%

Source: survey data.

Half of the CHWs were located in villages that were less than 5 km from a CSPS, which contradicted the plan. Only in 11 of the 26 villages were the communities actually involved in selecting the CHWs. This is largely due to the fact that the recruitment was based on coopting existing health workers. “*Since I was already working with the health workers, there was no mobilization of people to designate or vote for me*,” said one CHW in Zorgho. The CHWs “*existed before the HMM*,” a nurse in Kaya asserted. The fact that some of the older CHWs were unable to read or write explains why new CHWs had to be selected, sometimes only by a nurse: “*I selected them myself*.”

Problems with restocking ACTs occurred in two of Kaya’s 15 villages, compared with eight of the 13 villages in Zorgho, the difference being due particularly to the existence of multiple interventions in Kaya, as described above. The problem of remuneration remained significant in Zorgho, as the majority of CHWs reported not having been paid during their retraining, and seven out of 14 said they had not received any of the profits from the sales of ACTs. Likewise, many reported not having been paid during training and when distributing LLINs. In Kaya, these difficulties did not arise, except with regard to profits from ACT sales, which was problematic for six out of 15 of the CHWs for the reasons mentioned earlier, even though they had a good understanding of the principle of cost-recovery: “*this system is there to motivate us, since they’re not able to give us a salary*,” said one CHW in Kaya. On the whole, the monthly stipends for CHWs and NGO organizers were paid, but always late and covering several months at a time.

### Schedule Fidelity

The list of activities showed that few (six out of 47, or 13%) had explicit implementation schedules that would have allowed us to assess whether they were respected. With regard to the LLIN, we observed a delay of only a few weeks in the recruitment of census-takers, their training, and the conduct of the census itself. While they were all supposed to start in July 2010, their implementation was spread out over July and August 2010 in both districts. The greatest difficulty was in the distribution of LLINs, which was planned for July 2010 and finally only occurred in September (for three CSPSs) and October 2010 (for a fourth one), and in January 2011 in Kaya (for all the CSPSs); that was between two and six months late, and in each case, after the malaria season. With regard to HMM activities, the situation in Kaya was particular, because this district had been one of the three pilot districts for the NMCP. Thus, all the activities for which start dates had been planned were launched on those specified dates throughout the country. In other words, HMM was already in place in Kaya when the NMCP was launched. On the other hand, in Zorgho all the activities were delayed: recruitment of NGO organizers (two to 15 months, depending on the CSPSs), training of NHMs (one month), recruitment of CHWs (two months), training of CHWs (two to five months), initial stocking of ACTs (three months, starting after the malaria season, in October 2010).

## Discussion

Since this study involved only two districts, the results cannot be extrapolated to the entire country. Moreover, while the two cases are very similar, they are not wholly comparable in terms of their implementation and duration, their operational support, and parallel interventions. However, the use of two case studies where the program was implemented by two different NGOs reinforces the implementation’s replication logic and analytic generalization [27]. Memory biases were observed in certain participants, although it was not possible to know, particularly when the topic had to do with monetary aspects, whether these were true memory lapses or intentional behaviours. In the future, it would be important to understand why 12% to 17% of the activities were modified by the implementers. The modification of these activities was not, in itself, a problem [10], [24]; it may simply have been that the stakeholders wished to adapt them to their contexts for greater effectiveness, and without necessarily respecting the original plan and the implementation instructions to the letter. These adaptive strategies or, as some have analyzed them, reinventions [25], [30], remain to be studied.

Stakeholders’ responsiveness and facilitation strategies for program implementation are known to be moderating factors for implementation fidelity [24]. Thus, each CHW underwent three days of training on themes related to malaria (etiology, prevention, management) and on group facilitation in communities. Their knowledge was tested before and after the training. However, as in other programs in Burkina Faso [31], the MEILUP encountered obstacles related to these two subjects that could have modified its implementation fidelity. Thus, only some of them went through refresher sessions. Supervision was limited to sporadic visits by facilitators and did not include any meetings among CHWs to share their experiences, nor any monitoring by committees of community members, even though these two strategies were found to be useful elsewhere [32]. Furthermore, most of the CHWs recruited for the HMM had already occupied this position in other, earlier programs (path dependency) that were not found to be effective [33]. Many of them had been CHWs for more than 20 years, without really having the means to respond to the populations’ needs. Consequently, they may embody the failures of these past programs, which could dampen their enthusiasm for carrying out their functions and make them less appealing to the population. On the other hand, it should be noted that the CHWs’ remuneration (5000 F CFA per month) differs from previous practice, which consisted of providing occasional per diems; this might facilitate the implementation of program activities, as has been observed in other settings [34]. These factors that could modify the MEILUP’s implementation fidelity are present in Kaya and in Zorgho.

On the whole, we must conclude that the program’s implementation was relatively faithful to what was originally intended. No activity essential for the program’s effectiveness appears to have been omitted (i.e., availability of LLINs, initial distribution of ACTs, the presence of CHWs), and the effectiveness itself will be studied as part of the impact evaluation currently under way. However, from the results of this study, we can foresee a possible time lag in the appearance of the program’s effects. Indeed, the study highlighted difficulties in respecting the program’s coverage and schedule. The late, chaotic and reduced distribution of LLINs, the recurrent problems in restocking ACTs (which appear to have worsened since the data collection period), and regular stock shortages all raise serious concerns about the program’s sustainability and population effectiveness. Considering the combination of these factors and the fact that planned activities were delayed until past the height of the malaria season, it is unlikely that the program was able to achieve its objective of preventing some of the 40,000 malaria-related deaths in its first year of operation, 2010. The impact study currently under way may provide information on this subject.

It is nothing new that, as happened with the program for disease vector control in Cuba [25], activities that raise resource allocation issues would present difficulties in Burkina Faso, as shown recently in a parliamentary commission [35]. At the time when this program was launched, many people pointed to problems with respecting the procurement rules for LLINs. This situation certainly explains why an NGO was one of the two main recipients of this program. In fact, even before this scaling-up, the NMCP never seemed to have enough resources of its own to distribute mosquito nets, and the inputs were always provided by the Global Fund or other partners [35]. This raises questions as to whether the Global Fund’s principle of contributing additional resources to States, not replacement resources, was respected, in contrast to what happened in Timor, for example [36]. Stock shortages of LLINs and ACTs had already been observed before this scaling-up. At the end of 2010, for example, the CHWs of one of the pilot districts (Nouna) had still not received any ACTs, even though the scaling-up had started [14]. However, given that vertical programs endure in healthcare systems, whereas integration is one of the success factors in scaling up [37], [38], we might wonder whether this new program has not, once again, confirmed the need to pay more attention to issues of sustainability. Indeed, the WHO has pointed out that “*the rapid scale-up of ITN distribution in Africa is an enormous public health achievement, but also presents a formidable challenge for the future in ensuring that the levels of coverage are maintained”* [8]. In fact, the two essential components of perpetuation–the process leading to sustainability–are resource stability and organizational risk-taking [39].

In depending on resources from the Global Fund and relying on NGOs instead of the Ministry of Health for the HMM activities, the program’s chances of achieving sustainability are slim. The LLINs, ACTs and monthly stipends for CHWs are all funded by the Global Fund. The program is not sufficiently integrated into the health system, as was the case elsewhere where NGOs were used [36], [40]. The Ministry of Health’s nurses rarely supervise CHWs, which is not new in Burkina Faso [33], whereas such ongoing supervision is essential to ensure both the quality of the CHWs’ work, as was shown in Zambia [41], and integration into the health system [37]. Stock shortages of ACTs occur regularly and persistently. A national evaluation confirmed our results, showing that more that half the CHWs and the generic essential drug depots had experienced stock shortages during the previous six months in 26 of the country’s districts [42]. The authors of that report in fact pointed out that ACT shortages had been a recurrent phenomenon in the country for several years. Moreover, those in charge of the program wanted to organize a cost-recovery system for ACTs by selling them to families, which would not ensure stock sustainability and would provide only a meagre income to the CHWs ($0.05 per treatment), not to mention the incitement to prescription, as was seen in Senegal even in cases of negative rapid diagnoses [43]. As well, imposing such a financial barrier reduces access to treatment, particularly for the worst-off, as has been clearly shown in Burkina Faso in one of the three pilot districts [14] and in Mali [40].

Finally, in Kaya District, subsequent interventions authorized by the Ministry of Health interfered with the program. The Ministry of Health, funded by the Bill & Melinda Gates Foundation, and an international NGO funded by the European Union turned the program on its head. The former asked the CHWs, who were sometimes already working in the program, to take on the added job of treating children for diarrhea with rehydration salts and zinc. The second intervention had even more disruptive effects, as it organized a district-wide exemption from user fees for children under age five. Thus, since the program had chosen to subsidize ACTs rather than to eliminate payment, the CHWs’ work disappeared as mothers opted to bring their children to CSPSs for free care. This intervention was clearly not harmful to children’s health–in fact, quite the contrary–but it showed how a lack of cohesion and coordination can be damaging, in this case to the CHWs. Yet coordination of activities at the community level is one of the Ministry of Health’s priority actions [44]. A national evaluation deplored the systematic involvement of CHWs in the HMM program nationally without having first evaluated it in the pilot project in three districts [42]. This situation is particularly surprising given that CHWs have existed in Burkina Faso since the 1980s and that, despite their failings [33], the Ministry of Health decided in 2011 to create a new Department of Community Health largely consecrated to CHWs. However, the status of CHWs in the health system has not yet been resolved, creating challenges for their integration into the health system and the fight against malaria, as has been clearly shown in this study in Burkina Faso and was also seen in Senegal, for example [43]. The new national community health policy planned for 2012 should tell us more. Meanwhile, the resources allocated for CHWs’ training and monthly stipend are lost, since the CHWs no longer have work. A study is currently under way to better understand the effects of these multiple programs on CHWs, and particularly on mechanisms for their effectiveness [45].

Combining the implementation evaluation frameworks suggested by Carroll et al. [24] and Perez et al. [25] was fruitful. The latter was useful for extending beyond the former’s binary measurement of the implementation, by taking into account the occasional introduction of innovations by the stakeholders, which appears to have been more the norm than the exception in actual practice. Nevertheless, further conceptual work is needed to guide the coding of the radical or minor nature of certain components modified in the intervention, which was sometimes difficult to do in our analysis. Collecting information on minor changes was not always useful for understanding the implementation. A preliminary study with stakeholders might help to identify the essential and compulsory nature of certain activities before evaluating fidelity. Moreover, implementing an activity by the book may also show a lack of inventiveness. An example in our case was that of giving a bicycle to a CHW in order to respect the plan, when the CHW already had one; here, some adaptation would probably have been appropriate. Also, adding a fifth evaluation modality (implemented or modified) to the framework of Perez et al. [25] was useful in our specific context. In any qualitative study attempting to link implementation with outcomes, weighting an intervention’s components and calculating the extent of fidelity remain major challenges, which some have tried to address with methodological experiments using an implementation index [46].

Finally, our study also shows the need to take contextual dimensions and concurrent interventions more fully into account when studying fidelity of implementation. In fact, even though most FOI analysis frameworks consider context as a dimension to be studied [47], there has been very little in-depth research into this issue. For example, context is a very implicit dimension in Carroll’s framework [24], currently the most widely used, and for that reason was explicitly added to a proposal and later use by Hasson [26], [48]. As well, researchers also need to pay attention to the presence of other interventions running in parallel to the one whose implementation fidelity they are studying. These are part of the context are therefore also play a part in explaining the implementation. In the social arena, interventions are by nature complex and not implemented in a vacuum, and one feature of this complexity is the presence of overlapping interventions that some consider to be rivals [49]. In the context of our evaluation, it is essential to consider this rivalry of interventions, in that the action processes can interfere with each other, they use many of the same outreach workers (the same CHWs can work for both the HMM and BMG projects), they target largely overlapping populations (particularly children under 5), and ultimately contribute to meeting the same health needs. We will be studying this overlap in a future study but, on one hand, we can already say that the BMG intervention can be expected to enhance the HMM implementation because it also reinforced the CHWs’ knowledge and sent out a strong message to the population that CHWs play a useful role in managing malaria. However, on the other hand, a new NGO started an intervention in the Kayes district in 2011 that eliminated user fees in health centres for children under 5 years, which could greatly undermine the role of CHWs, who still charge fees for ACTs in villages. Current fidelity of implementation analysis frameworks do not sufficiently take into account these overlapping actions and the presence of concurrent projects [48]. This may be because there have not yet been enough studies on implementation in Africa [21]. It could also be that the strong presence of public development aid in this context means it is also where the potential for multiple interventions is greatest, given the difficulties that States have had in coordinating public policies with externally funded interventions, regardless of the operational levels involved. It was this situation that prompted the Paris Declaration [50] for better harmonization of interventions and their alignment with national priorities. Our study context shows that this does not yet appear to be entirely the case, a situation which merits further analysis.

### Conclusions

The implementation analysis of this program in Burkina Faso showed that the activities were, on the whole, relatively faithful to the plan. However, delays in the implementation schedule and problems related to LLIN and ACT distribution definitely postponed the potential beneficial effects of this malaria control effort. The impact analysis should help to shed light on this question.

## Supporting Information

Table S1 **Content fidelity of activities in Kaya District.** Source: survey data.
(PDF)

Table S2 **Content fidelity of activities in Zorgho District.** Source: survey data.
(PDF)

Table S3 **Coverage fidelity of activities in Kaya District.** Source: survey data.
(PDF)

Table S4 **Coverage fidelity of activities in Zorgho District.** Source: survey data.
(PDF)
