# Supplementary material for: Implementation Fidelity of the National Malaria Control Program in Burkina Faso
Source: PLoS One. 2013 Jul 26;8(7):e69865. doi: 10.1371/journal.pone.0069865 (PMC3724672; doi:10.1371/journal.pone.0069865)
Supplement: Table S1 — Content fidelity of activities in Kaya District. Source: survey data. (PDF) [file pone.0069865.s001.pdf]

Table S1 Content fidelity of activities in Kaya District

		CSPS1	CSPS2	CSPS3	CSPS4	CSPS5	CSPS6
**LLIN**							
Recruitment and training							
	Census-takers recruited	I	I	I	I	I	I
	Census-takers trained (1 day)	M	I	I	I	M	M
	NHMs trained for the census (2 days)	I/M	M	M	M	I/M	M
	NGO organizers trained for the census (2 days)	I/M	I/M	I/M	I/M	I/M	I
Activities							
	Distribution of LLINs	I	I	I	I	I	I
	Follow-up of LLIN use by CHWs and NGO organizers	I	I	I	I	I	I
	Census-taking	I	I	I	I	I	I
Remuneration							
	CHWs remunerated for training	M	I/M	I	I/M	I/M	I/M
	CHWs remunerated for census-taking	I	I and M	I	I/M	I/M	I
	CHWs remunerated for distribution	I	I and M	I	M	I/M	(NA)
	NHMs remunerated for training	I	I	I	M	I or M	I
	NGO organizers remunerated for training	I/M	I/M	I/M	I	I	I
**HMM**							
Recruitment and training							

	Recruitment of a CHW by the village	I	I	I	I	I	I
	Community participation in CHW selection	N	I and N		I	I	N
	Recruitment of an NGO organizer	I	I	I	I	I	I
	Training of CHWs (3 days)	M	I and M	M	M	M	I and M
	Retraining of CHWs	A	A and I	I	A	A	A
	Training of NGO organizers (2 days)	I/M	I/M	I/M	M	M	M
	Training of NHMs (2 days)	I/M	I/M	I	I	I/M	I/M
Provision of materials							
	I carrying case per CHW	I	N	N	N	N	I
	Initial stock for the CHWs	M	M	I	M	I and M	I
	Replenishment of the CHWs' ACT stocks	M	M	M	M	M	M
	1 collection book per CHW	I	I	I	I	N	I
	1 consultation register per CHW	M	M	M	M	N	M
	1 training module	I	I	I	I	I	I
	Stocking forms	N	N	N	N	N	N
	1 box of images per CHW	A	A	A	A	A	A
	1 bicycle per CHW	I	I	I	I	I	I
	Audiovisual materials for the NGO organizers	N	N	N	N	N	N
	1 motorbike (with helmet) for each NGO organizer	I	I	I	I	I	I
Activities							
	3 HV per month (CHW)	I	I	I	I	I	I

	1 educational talk per month (CHW)	I	I	I	I	I	I
	Supervision of CHWs (NGO organizers)	I	I	I	I	I	I
	Skits and film projections (NGO organizers)	N	N	N	N	N	N
	2 talks per month per village (NGO organizers)	I	I	I	I	I	I
	2 HV per month per village (NGO organizers)	I	I	I	I	I	I
	2 co-facilitated sessions per month per village (NGO organizers + CHWs)	I	I	I	N	N	N
	Approval of the CHWs’ monthly reports (NHM)	N	I	I	I	N	I
	Approval of the NGO organizers’ monthly program (NHM)	I	I	I	I	I	I
Remuneration							
	CHWs remunerated for training	M	M	I/M	M	M	M
	CHWs remunerated for retraining	A	A	(NA)	A	A	A
	NGO organizers remunerated for training	I/M	I/M	I/M	I	I	I
	NHMs remunerated for training	I	I/M	I	I/M	I/M	I
	Monthly stipend for CHWs	I	I	I	I	I	M
	Profit on ACT sales	I	I	N	I and N	N	I
	Monthly stipend for NGO organizers	I	I	I	I	I	I

	Allocations for NGO organizers' travel costs	M	M	M	M	M	M

Note: CHW = community health workers; NHM = nurse health-post manager; ACT = artemisinin-combination therapy; HV = home visit; I = implemented as intended; I/M = implemented or modified; M = modified; A = added; N = not implemented; (NA) = not applicable.
