# Supplementary material for: Implementation Fidelity of the National Malaria Control Program in Burkina Faso
Source: PLoS One. 2013 Jul 26;8(7):e69865. doi: 10.1371/journal.pone.0069865 (PMC3724672; doi:10.1371/journal.pone.0069865)
Supplement: Table S2 — Content fidelity of activities in Zorgho District. Source: survey data. (PDF) [file pone.0069865.s002.pdf]

Table S2 Content fidelity of activities in Zorgho District

		CSPS1	CSPS2	CSPS3	CSPS4	CSPS5
**LLIN**						
Recruitment and training						
	Census-takers recruited	I	I	I	I	I
	Census-takers trained (1 day)	I/M	M	I	I	I
	NHMs trained for the census (2 days)	I/M	M	N	I	I
	NGO organizers trained for the census (2 days)	I/M	(NA)	I	N	N
Activities						
	Distribution of LLINs	I	I	I	I	I
	Follow-up of LLIN use by CHWs and NGO organizers	I	I	I	I	I
	Census-taking	I	I	I	I	I
Remuneration						
	CHWs remunerated for training	N	N	I	M	N
	CHWs remunerated for census-taking	N	I/M	I	M	M
	CHWs remunerated for distribution	N	N	I	I/M	N
	NHMs remunerated for training	I/M	M	N	I/M	I/M
	NGO organizers remunerated for training	I/M	(NA)	N	(NA)	(NA)
**HMM**						
Recruitment and training						
	Recruitment of a CHW by the village	I	I	I	I	I
	Community participation in CHW selection	I and N	I	I	N	I and N
	Recruitment of an NGO organizer	I	I	I	I	I

	Training of CHWs (3 days)	I and M	I	I	M	I
	Retraining of CHWs	A and I	A	A	A	A and I
	Training of NGO organizers (2 days)	I	M	I	N	N
	Training of NHMs (2 days)	I/M	M	I	M	I
Provision of materials						
	I carrying case per CHW	I	I	I	I	I
	Initial stock for the CHWs	I	I	I	I	I
	Replenishment of the CHWs' ACT stocks	N	N	I	(NA)	N
	1 collection book per CHW	I	I	I	I	I
	1 consultation register per CHW	N	N	N	N	N
	1 training module	N	I	I	I	N
	Stocking forms	I	I	I	I	I
	1 box of images per CHW	A and I	N	A	A	N
	1 bicycle per CHW	I	I	I	I	I
	Audiovisual materials for the NGO organizers	N	N	N	N	N
	1 motorbike (with helmet) for each NGO organizer	I	I	I	I	I
Activities						
	3 HV per month (CHW)	I	I	I	I	I
	1 educational talk per month (CHW)	I	I	I	I	I
	Supervision of CHWs (NGO organizers)	I	I	I	I	I
	Skits and film projections (NGO organizers)	N	N	N	N	N
	2 talks per month per village (NGO organizers)	I	I	I	I	I
	2 HV per month per village (NGO	I	I	I	I	I

	organizers)					
	2 co-facilitated sessions per month per village (NGO organizers + CHWs)	I	I	I	M	M
	Approval of the CHWs' monthly reports (NHM)	N	N	I	N	N
	Approval of the NGO organizers' monthly program (NHM)	I	I	I	I	I
Remuneration						
	CHWs remunerated for training	I and M	I/M	I/M	I	I/M
	CHWs remunerated for retraining	I	I	I	A	I
	NGO organizers remunerated for training	I	N	M	(NA)	(NA)
	NHMs remunerated for training	I/M	I	M	I	I
	Monthly stipend for CHWs	I	I	I	I	I
	Profit on ACT sales	I and N	I	I	I	I and N
	Monthly stipend for NGO organizers	M	M	M	M	M
	Allocations for NGO organizers' travel costs	M	M	M	M	M

Note: CHW = community health workers; NHM = nurse health-post manager; ACT = artemisinin-combination therapy; HV = home visit; I = implemented as intended; I/M = implemented or modified; M = modified; A = added; N = not implemented; (NA) = not applicable.
