# Supplementary material for: Implementation Fidelity of the National Malaria Control Program in Burkina Faso
Source: PLoS One. 2013 Jul 26;8(7):e69865. doi: 10.1371/journal.pone.0069865 (PMC3724672; doi:10.1371/journal.pone.0069865)
Supplement: Table S3 — Coverage fidelity of activities in Kaya District. Source: survey data. (PDF) [file pone.0069865.s003.pdf]

Table S3 Coverage fidelity of activities in Kaya District

		CSPS1	CSPS2	CSPS3	CSPS4	CSPS5	CSPS6	Total
**LLIN**								
Recruitment and training								
	CHWs trained as census-takers	1/1	5/5	1/3	2/2	2/2	2/2	87%
	NHMs trained for the census	1/1	1/1	1/1	1/1	1/1	1/1	100%
	NGO organizers trained for the census	1/1	1/1	1/1	1/1	1/1	1/1	100%
Activities								
	Follow-up of LLIN use in the villages by the CHWs and NGO organizers	1/2	5/6	3/5	2/2	2/2	2/2	79%
	Household census	98%	90 % or +	90% or +	100%	99%	100%	96%
Remuneration								
	CHWs remunerated for training	1/1	5/5	1/1	2/2	2/2	2/2	100%
	CHWs remunerated for census-taking	1/1	4/5	1/1	2/2	2/2	2/2	92%
	CHWs remunerated for distribution	1/1	4/5	1/1	2/2	2/2	(NA)	91%
	NHMs remunerated for training	1/1	1/1	1/1	1/1	1/1	1/1	100%
	NGO organizers remunerated for training	1/1	1/1	1/1	1/1	1/1	1/1	100%
**HMM**								
Recruitment and training								
	Appropriate geographic coverage of HMM CHWs	1/2	5/6	3/5	1/2	0/2	0/2	53%

	CHWs trained (3 days)	1/1	5/5	2/3	2/2	2/2	2/2	93%
	Community involvement in selecting CHWs	0/1	1/5		2/2	2/2	0/2	42%
	CHWs retrained	1/1	4/5	0/3	2/2	2/2	2/2	73%
	NGO organizers trained (2 days)	1/1	1/1	1/1	1/1	1/1	1/1	100%
	NHMs trained (2 days)	1/1	1/1	1/1	1/1	1/1	1/1	100%
Provision of materials								
	1 carrying case per CHW	1/1	0/5	0/3	0/2	0/2	2/2	20%
	Initial stock for the CHWs	1/1	5/5	1/3	2/2	2/2	2/2	87%
	Replenishment of the CHWs' ACT stock	1/1	5/5	3/3	2/2	1/2	2/2	87%
	I collection book per CHW	1/1	5/5	3/3	2/2	0/2	2/2	87%
	I consultation register per CHW	1/1	5/5	3/3	2/2	1/2	2/2	93%
	I training module per CHW	1/1	5/5	3/3	2/2	2/2	2/2	100%
	1 stocking form per CHW	0/1	0/5	0/3	0/2	0/2	0/2	0%
	1 box of images per CHW	1/1	5/5	3/3	2/2	2/2	2/2	100%
	1 bicycle per CHW	1/1	5/5	3/3	2/2	2/2	2/2	100%
	Audiovisual materials for the NGO organizers	0/1	0/1	0/1	0/1	0/1	0/1	0%
	1 motorbike (with helmet) for each NGO organizer	1/1	1/1	1/1	1/1	1/1	1/1	100%
Activities								
	3 HV per month (CHW)	1/1	5/5	3/3	2/2	2/2	2/2	100%
	1 educational talk per month (CHW)	1	1	1	1	1	1	100%
	Supervision of CHWs (NGO	1	1	1	1	1	1	100%

	organizers)							
	Skits and film projections (NGO organizers)	0	0	0	0	0	0	0%
	2 talks per month per village (NGO organizers)	1	1	1	1	1	1	100%
	2 HV per month per village (NGO organizers)	1	1	1	1	1	1	100%
	2 co-facilitated sessions per month per village (NGO organizers + CHWs)	1/1	5/5	3/3	0/2	0/2	0/2	60%
	Approval of the CHWs' monthly reports (NHM)	0/1	5/5	3/3	2/2	1/2	2/2	87%
	Approval of the NGO organizers' monthly program (NHM)	1	1	1	1	1	1	100%
Remuneration								
	CHWs remunerated for training	1	1	1	1	1	1	100%
	CHWs remunerated for retraining	1	1	(NA)	1	1	1	100%
	NGO organizers remunerated for training	1	1	1	1	1	1	100%
	NHMs remunerated for training	1	1	1	1	1	1	100%
	Monthly stipend for CHWs	1	1	1	1	1	1	100%
	Profit on ACT sales	1/1	5/5	0/3	1/2	0/2	2/2	60%
	Monthly stipend for NGO organizers	1	1	1	1	1	1	100%
	Allocations for NGO organizers'	1	1	1	1	1	1	100%

	travel costs							

Note: CHW = community health workers; NHM = nurse health-post manager; ACT = artemisinin-combination therapy; HV = home visit; (NA) = not applicable.
