# Supplementary material for: Implementation Fidelity of the National Malaria Control Program in Burkina Faso
Source: PLoS One. 2013 Jul 26;8(7):e69865. doi: 10.1371/journal.pone.0069865 (PMC3724672; doi:10.1371/journal.pone.0069865)
Supplement: Table S4 — Coverage fidelity of activities in Zorgho District. Source: survey data. (PDF) [file pone.0069865.s004.pdf]

Table S4 Coverage fidelity of activities in Zorgho District

		CSPS1	CSPS2	CSPS3	CSPS4	CSPS5	Total
**MILDA**							
Recruitment and training							
	CHWs trained as census-takers	6/6	2/2	1/1	1/1	3/4	93%
	NHMs trained for the census	1/1	1/1	0/1	1/1	1/1	80%
	NGO rganizers trained for the census	1/1	0/1	1/1	0/1	0/1	40%
Activities							
	Follow-up of LLIN use in the villages by the CHWs and NGO organizers	6/7	2/3	1/2	1/1	4/6	74%
	Household census	100%	100%	100%	100%	100%	100%
Remuneration							
	CHWs remunerated for training	1/6	0/2	1/1	1/1	2/3	38%
	CHWs remunerated for census-taking	4/6	2/2	1/1	1/1	3/3	85%
	CHWs remunerated for distribution	2/6	0/2	1/1	1/1	1/2	42%
	NHMs remunerated for training	1/1	1/1	(NA)	1/1	1/1	100%
	NGO organizers remunerated for training	1/1	(NA)	0/1	(NA)	(NA)	50%
**HMM**							
Recruitment and training							
	Appropriate geographic coverage of HMM CHWs	5/7	3/3	2/2	0/1	5/6	79%
	CHWs trained (3 days)	6/6	2/2	1/1	1/1	4/4	100%

	Community involvement in selecting CHWs	3/6	2/2	0/1	0/1	1/4	43%
	CHWs retrained	5/6	2/2	1/1	1/1	2/4	79%
	NGO organizers trained (2 days)	1/1	1/1	1/1	0/1	0/1	60%
	NHMs trained (2 days)	1/1	1/1	1/1	1/1	1/1	100%
Provision of materials							
	1 carrying case per CHW	6/6	2/2	1/1	1/1	4/4	100%
	Initial stock for the CHWs	6/6	2/2	1/1	1/1	4/4	100%
	Replenishment of the CHWs' ACT stock	4/6	0/2	1/1	(NA)	0/4	38%
	I collection book per CHW	6/6	2/2	1/1	1/1	4/4	100%
	I consultation register per CHW	0/6	0/2	0/1	0/1	0/4	0%
	I training module per CHW	4/6	2/2	1/1	1/1	2/4	71%
	1 stocking form per CHW	6/6	2/2	1/1	1/1	4/4	100%
	1 box of images per CHW	5/6	0/2	1/1	1/1	0/4	50%
	1 bicycle per CHW	6/6	2/2	1/1	1/1	4/4	100%
	Audiovisual materials for the NGO organizers	0/1	0/1	0/1	0/1	0/1	0%
	1 motorbike (with helmet) for each NGO organizer	1/1	1/1	1/1	1/1	1/1	100%
Activities							
	3 HV per month (CHW)	6/6	2/2	1/1	1/1	4/4	100%
	1 educational talk per month (CHW)	6/6	2/2	1/1	1/1	4/4	100%
	Supervision of CHWs (NGO organizers)	6/6	2/2	1/1	1/1	4/4	100%
	Skits and film projections (NGO	0/1	0/1	0/1	0/1	0/1	0%

	organizers)						
	2 talks per month per village (NGO organizers)	1/1	1/1	1/1	1/1	1/1	100%
	2 HV per month per village (NGO organizers)	1/1	1/1	1/1	1/1	1/1	100%
	2 co-facilitated sessions per month per village (NGO organizers + CHWs)	1/1	1/1	1/1	0/1	0/1	60%
	Approval of the CHWs' monthly reports (NHM)	0/1	0/1	1/1	0/1	0/1	20%
	Approval of the NGO organizers' monthly program (NHM)	1/1	1/1	1/1	1/1	1/1	100%
Remuneration							
	CHWs remunerated for training	6/6	2/2	1/1	1/1	4/4	100%
	CHWs remunerated for retraining	0/6	0/2	0/1	1/1	0/2	8%
	NGO organizers remunerated for training	1/1	0/1	1/1	(NA)	(NA)	67%
	NHMs remunerated for training	1/1	1/1	1/1	1/1	1/1	80%
	Monthly stipend for CHWs	6/6	2/2	1/1	1/1	4/4	100%
	Profit on ACT sales	1/6	2/2	1/1	1/1	2/4	50%
	Monthly stipend for NGO organizers	1/1	1/1	1/1	1/1	1/1	100%
	Allocations for NGO organizers' travel costs	1/1	1/1	1/1	1/1	1/1	100%

Note: CHW = community health workers; NHM = nurse health-post manager; ACT = artemisinin-combination therapy; HV = home visit; (NA): not applicable.
